# Seasonality Impact on the Transmission Dynamics of Tuberculosis

**DOI:** 10.1155/2016/8713924

**Published:** 2016-03-02

**Authors:** Yali Yang, Chenping Guo, Luju Liu, Tianhua Zhang, Weiping Liu

**Affiliations:** ^1^Science College, Air Force Engineering University, Xi'an, Shaanxi 710051, China; ^2^College of Mathematics and Information Science, Shaanxi Normal University, Xi'an, Shaanxi 710062, China; ^3^Centre for Disease Modelling, York University, Toronto, ON, Canada M3J 1P3; ^4^School of Mathematics and Statistics, Henan University of Science and Technology, Luoyang 471023, China; ^5^Institute of Tuberculosis Prevention and Treatment in Shaanxi, Xi'an, Shaanxi 710048, China

## Abstract

The statistical data of monthly pulmonary tuberculosis (TB) incidence cases from January 2004 to December 2012 show the seasonality fluctuations in Shaanxi of China. A seasonality TB epidemic model with periodic varying contact rate, reactivation rate, and disease-induced death rate is proposed to explore the impact of seasonality on the transmission dynamics of TB. Simulations show that the basic reproduction number of time-averaged autonomous systems may underestimate or overestimate infection risks in some cases, which may be up to the value of period. The basic reproduction number of the seasonality model is appropriately given, which determines the extinction and uniform persistence of TB disease. If it is less than one, then the disease-free equilibrium is globally asymptotically stable; if it is greater than one, the system at least has a positive periodic solution and the disease will persist. Moreover, numerical simulations demonstrate these theorem results.

## 1. Introduction

Tuberculosis (TB) remains one of the world's deadliest communicable diseases. In 2013, it was estimated that 9.0 million people developed TB and 1.5 million died from the disease, and TB is slowly declining each year [1]. According to the online global TB data collection system, China alone accounted for 11% of the total cases, which is the second country of 22 TB high-burden countries, only after India. Shaanxi is one of the more serious TB provinces in China, and its reported new cases each year reach about 25,000, which is the second in the number of cases of infectious diseases in Shaanxi, only next to the hepatitis B virus (HBV).

Some researchers have investigated the influence of seasonal variations on the transmission dynamics of infectious diseases [2–4]. And seasonal variation in TB incidence has been described in many countries and cities, such as India, United States, Russia, New York city, and Hong Kong [5–9]. TB is a seasonal disease in China [10, 11], but it remains unknown in Shaanxi.

From 2004 to 2012, there are 273,305 reported notifiable active TB cases in Shaanxi. Monthly reports of notifiable active TB cases from January 2004 to December 2012 in Shaanxi (Table 1) are available on the data-center of China public health science [12]. We apply seasonal filters in MATLAB program, to deseasonalize the time series; then the original time series is decomposed into three components: trend curve and season and irregular noise. The trend curve is the long-term and medium-to-long term movement of the series; it also contains consequential turning points; the seasonal component is within one-year (12 months) fluctuations about the trend that recur in a similar way in the same month or quarter every year; and the irregular component is the residual component that still remains after trend curve and seasonal component are removed from the original series.

**Figure 1:**
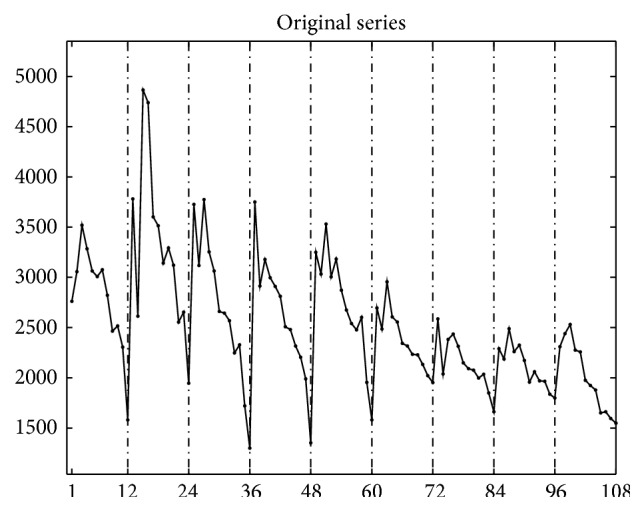
The original time series of pulmonary TB cases in Shaanxi of China, January 2004 to December 2012.

**Figure 2:**
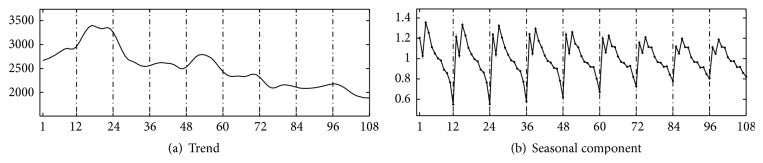
(a) The trend component of time series for pulmonary TB cases in Shaanxi of China, January 2004 to December 2012. (b) The seasonal component of time series for pulmonary TB cases in Shaanxi of China, January 2004 to December 2012.

**Figure 3:**
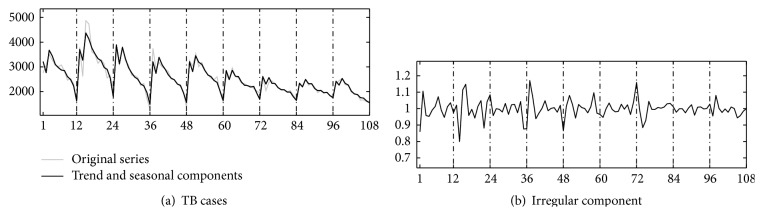
(a) Compare the original series to a series reconstructed using the component estimates of time series for pulmonary TB cases in Shaanxi of China, January 2004 to December 2012. (b) The irregular noise component of time series for pulmonary TB cases in Shaanxi of China, January 2004 to December 2012.

Figure 1 shows the original time series of active TB cases from January 2004 to December 2012 in Shaanxi. Figures 2(a) and 2(b) show the isolated trend curve and the seasonal component, respectively. To show the correctness of this multiplicative decomposition, the original series are compared to a series reconstructed using the component estimates in Figure 3(a) and giving irregular noise component in Figure 3(b). It has shown that the multiplicative decomposition is fit for the TB data of Shaanxi.

In Figure 2(a), trend component shows that there is a fast upward trend after 2004 and in 2005 (the data in 2004 is low just because this is the first year for reported data, and some staff may just begin to the report system, so some patients may be omitted), then a downward trend from 2005 to 2007, and then a slowly upward in 2008, a steadily decreasing trend from 2009 to 2012. In Figure 2(b), firstly, seasonal component shows that seasonal amplitude decreases each year from 2004 to 2012; secondly, it illustrates there exists a seasonal period *T*, 12 months, and along with peak and trough months: the first- and second-peak month of TB notification in March and January, respectively, and between them, there exists a trough, February, between these two months (February may be a peak month, but for Chinese lunar new year, Spring Festival, there exists a special reason that patients may not choose diagnosis for regarding illness as an unlucky thing in this traditional festival); the trough month is in December. TB is a seasonal disease in Shaanxi. In addition, the peak and trough of TB transmission actually are in winter and in autumn, respectively, due to the delay which tends to last 4–8 weeks. Understanding TB seasonality may help TB programs better to plan and allocate resources for TB control activities [8]. Motivated by this, we formulate a seasonality *SLIR* epidemic model with periodic coefficients for TB and study its global dynamics in this paper.

This paper is organized as follows. In Section 2, a seasonality TB model is formulated. In Section 3, a unique disease-free equilibrium is obtained, and the basic reproduction number *R*_*T*_ for the periodic model is given in detail. Furthermore, some numerical simulations are used to compare the average basic reproduction number [*R*_*T*_] to *R*_*T*_ in different cases. Threshold dynamics of the TB model is analyzed in Section 4. Meanwhile, some numerical simulations are provided to validate analytical results. Finally, conclusions are given in Section 5.

## 2. Model Formulation

The total population is divided into four compartments: susceptible (*S*), latent (*L*), infectious (*I*), and recovered (*R*) individuals.

**Figure 4:**
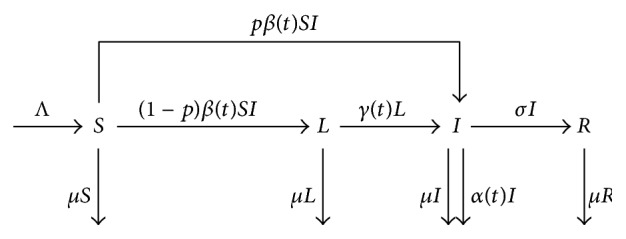
The transfer diagram for model (1).

From Figure 2(b), new TB cases have shown the periodic monthly trend and the possible causes of the seasonal pattern. Shaanxi has a continental monsoon climate and has four distinct seasons. Seasons in Shaanxi are defined as spring (February–April), summer (May–July), autumn (August–October), and winter (November–January). TB is usually acquired through airborne infection from active TB cases; its transmission and progress tend to be effected by the climate within one year (12 months). In particular, the indoor activities are much more in winter than in a warm climate, which improves the probability of susceptible individuals exposed to* Mycobacterium tuberculosis* (Mtb) from the infectious individuals in a room with windows closed for a longer period of time [13]; thus infection rate may have the periodic influence. In addition, during these months near or in the highest peak month of TB cases, cold weather and lack of sunshine, which may reduce human immunity, cause a higher disease-induced rate. Individuals with lower Vitamin D level may be more reactivated for TB [14]. Thus, disease-induced rate and reactivation rate may also have the periodic influence. To describe and study the TB transmission in Shaanxi, three periodic coefficients are selected: (i) infection rate coefficient *β*(*t*) and the bilinear incidence *β*(*t*)*SI* which are applied in this model; (ii) reactivation rate coefficient *γ*(*t*), at which an individual leaves the latent compartment for becoming infectious; and (iii) disease-induced rate coefficient *α*(*t*), which is the disease-induced death rate coefficients for individuals in compartment *I*. In view of the periodic trend of monthly, *β*(*t*), *γ*(*t*), and *α*(*t*) are assumed to be positive periodic continuous function of *t* with period *T*. In some TB models, the fast and slow progression has been considered [15–17]. Based on those, a seasonality TB model with fast and slow progression and periodic coefficients is formulated in this section. The transfer among compartments is schematically depicted in Figure 4. It leads to the following model of ordinary differential equations:

(1) S′=Λ−βtSI−μS,L′=1−pβtSI−γtL−μL,I′=pβtSI+γtL−σI−αtI−μI,R′=σI−μR,

with initial condition (*S*(0), *L*(0), *I*(0), *R*(0)) = (*S*_0_, *L*_0_, *I*_0_, *R*_0_) ∈ *ℝ*_+_^4^, and all parameters are positive. Here, Λ is the recruitment rate and parameter *μ* is the natural death rate coefficient; *σ* is the rate coefficient at which an infective individual leaves the infectious compartment to the recovered; *p*  (0 ≤ *p* < 1) is the fraction of infected individuals who are fast developing into infected cases and enter the infectious compartment directly, while 1 − *p* is the fraction of infected individuals who are slowly developing into infected cases and transferred to the latent compartment.

Since *R* does not appear in the other equations of system (1), system (1) is equivalent to the following system:

(2) S′=Λ−βtSI−μS,L′=1−pβtSI−γtL−μL,I′=pβtSI+γtL−σI−αtI−μI.

**Theorem 1 .**

Every forward solution (*S*(*t*), *L*(*t*), *I*(*t*)) of system (2) eventually enters *Ω* = {(*S*, *L*, *I*) ∈ *ℝ*_+_^3^ : *S* + *L* + *I* ≤ Λ/*μ*}, and *Ω* is a positively invariant set for system (2).

**Proof**

From system (1), it follows that

(3) S+L+I+R′Λ−μS+L+I+R−αtI≤Λ−μS+L+I+R,

and then

(4) $$\begin{array}{c} \underset{t\to \infty }{\mathrm{limsup}}\left(\;S+L+I+R\;\right)\leq \frac{\Lambda }{\mu }. \end{array}$$

It implies that region *X* = {(*S*, *L*, *I*, *R*) ∈ *ℝ*_+_^4^ : *S* + *L* + *I* + *R* ≤ Λ/*μ*} is a positively invariant set for system (1). Then, *Ω* = {(*S*, *L*, *I*) ∈ *ℝ*_+_^3^ : *S* + *L* + *I* ≤ Λ/*μ*} is a positive invariant with respect to system (2). Therefore, system (2) is dissipative, and its global attractor is contained in *Ω*.

In the rest of this paper system (2) will be studied in region *Ω*.

## 3. Disease-Free Equilibrium and the Basic Reproduction Number

To study system (2), some notations are introduced.

Let (*ℝ*, *ℝ*_+_^*n*^) be the standard ordered *n*-dimensional Euclidean space with a norm ‖·‖. For *u*, *v* ∈ *ℝ* ^*n*^, denote *u* ≥ *v* if *u* − *v* ∈ *ℝ*_+_^*n*^; *u* > *v* if *u* − *v* ∈ *ℝ*_+_^*n*^∖{0}; and *u* ≫ *v* if $u-v\in \underset{}{\mathrm{Int}}\left({\mathbb{R}}_{+}^{n}\right)$.

Let *A*(*t*) be a continuous, cooperative, irreducible, and *n* × *n* matrix function with period *T* > 0, and let Φ_*A*_(*t*) be the fundamental solution matrix of the linear ordinary differential equation:

(5) $$\begin{array}{c} \frac{dx}{dt}=A\left(\;t\;\right)x. \end{array}$$

And let *ρ*(Φ_*A*_(*T*)) be the spectral radius of Φ_*A*_(*T*). By Perron-Frobenius theorem, *ρ*(Φ_*A*_(*T*)) is the principle eigenvalue of Φ_*A*_(*T*), in the sense that it is simple and admits an eigenvector *v* ^*∗*^ ≫ 0.

There is a unique disease-free steady state *E*_0_, that is (Λ/*μ*, 0,0), for system (2).

In the following, the basic reproduction number *R*_*T*_ will be introduced for system (2) according to the general procedure presented in [2].

With *χ*≔(*L*, *I*, *S*), system (2) becomes

(6) $$\begin{array}{c} \chi^{\prime }\left(\;t\;\right)=F\left(\;\chi \;\right)-V\left(\;\chi \;\right), \end{array}$$

where

(7) Fχ=1−pβtSIpβtSI0,Vχ=γtL+μL−γtL+σI+αtI+μI−Λ+μS.

Furthermore, here comes

(8) Ft=01−pβtΛμ0pβtΛμ,Vt=γt+μ0−γtσ+αt+μ.

Then *F*(*t*) is nonnegative, and −*V*(*t*) is cooperative in the sense that the off-diagonal elements of −*V*(*t*) are nonnegative. Thus, it is easy to verify that system (2) satisfies the assumptions (A1)–(A7) in [2].

Define *Y*(*t*, *s*), *t* ≥ *s*, which is a 2 × 2 matrix, and is the evolution operator of the linear *T*-periodic system

(9) $$\begin{array}{c} \frac{dy}{dt}=-V\left(\;t\;\right)y. \end{array}$$

That is, for each *s* ∈ *ℝ*, *Y*(*t*, *s*) satisfies

(10) $$\begin{array}{c} \frac{dY\left(t,s\right)}{dt}=-V\left(\;t\;\right)Y\left(\;t,s\;\right),\;\forall t\geq s,\;\;Y\left(s,s\right)=E, \end{array}$$

where *E* is the 2 × 2 identity matrix. Thus, the monodromy matrix Φ_−*V*(*t*)_ of (9) equals *Y*(*t*, 0), *t* ≥ 0. Assume that the population is near the disease-free periodic state *E*_0_. And suppose that *ϕ*(*s*), *T*-periodic in *s*, is the initial distribution of infectious individuals. Then *F*(*s*)*ϕ*(*s*) is the rate of new infections produced by the infected individuals who were introduced at time *s*. Given *t* ≥ *s*, *Y*(*t*, *s*)*F*(*s*)*ϕ*(*s*) gives the distribution of those infected individuals who were newly infected at time *s* and remain in infected compartments at *t*. It follows that

(11) ψt∫−∞tYt,sFsϕsds=∫0∞Yt,t−aFt−aϕt−ada

is the distribution of accumulative new infections at time *t* produced by all those infected individuals *ϕ*(*s*) introduced at time *s*  (*s* ≤ *t*). Let *C*_*T*_ be the ordered Banach space of all *T*-periodic functions from *ℝ* to *ℝ* ^*n*^, which is equipped with the maximum norm ||·||_*∞*_ and the positive cone *C*_*T*_^+^ = {*ϕ* ∈ *C*_*T*_ : *ϕ*(*t*) ≥ 0,  *t* ∈ *ℝ*}. Define a linear operator *H* : *C*_*T*_ → *C*_*T*_ by

(12) Hϕt=∫0∞Yt,t−aFt−aϕt−ada,∀t∈R,  ϕ∈CT.

Then, according to Wang and Zhao [2], the basic reproduction number *R*_*T*_ is defined as

(13) $$\begin{array}{c} R_{T}≔\rho \left(\;H\;\right) \end{array}$$

for the periodic epidemic system (2), where *ρ*(*H*) denotes the spectral radius of the matrix *H*.

In the constant case, that is, *β*(*t*) ≡ *β*, *γ*(*t*) ≡ *γ*, *δ*(*t*) ≡ *δ*, ∀*t* > 0, then *F*(*t*) ≡ *F*, *V*(*t*) ≡ *V*, ∀*t* > 0, in which

(14) F=01−pβΛμ0pβΛμ,V=γ+μ0−γσ+α+μ.

By van den Driessche and Watmough [18], here comes

(15) $$\begin{array}{c} R_{T}=\rho \left(\;FV^{-1}\;\right)=\frac{\beta }{\mu +\alpha +\sigma }\left(\;p+\frac{\gamma }{\mu +\gamma }\left(\;1-p\;\right)\;\right). \end{array}$$

In the periodic case, in order to characterize *R*_*T*_, consider the following linear *T*-periodic equation:

(16) $$\begin{array}{c} \frac{dw}{dt}=\left(\;-V\left(\;t\;\right)+\frac{F\left(t\right)}{\lambda }\;\right)w,\;\forall t\in R, \end{array}$$

with parameter *λ* ∈ (0, *∞*). Let *W*(*t*, *s*, *λ*) be the evolution operator of system (16) on *ℝ* ^2^, and *R*_*T*_ can be calculated in numerically according to Lemma 2.

**Lemma 2 (Wang and Zhao, [2], Theorem 2.1).**

For system (2), the following statements are valid:

(i) If *ρ*(*W*(*T*, 0, *λ*)) = 1 has a positive solution *λ*_0_, then *λ*_0_ is an eigenvalue of *H*, and hence *R*_*T*_ > 0.
(ii) If *R*_*T*_ > 0, then *λ* = *R*_*T*_ is the unique solution of *ρ*(*W*(*T*, 0, *λ*)) = 1.
(iii) *R*_*T*_ = 0 if and only if *ρ*(*W*(*T*, 0, *λ*)) < 1, ∀*λ* > 0.

And the threshold behaviors occur.

**Lemma 3 (Wang and Zhao [2], Theorem 2.2).**

For system (2), the following statements are valid:

(i) *R*_*T*_ = 1 if and only if *ρ*(Φ_*F*(*t*)−*V*(*t*)_(*T*)) = 1.
(ii) *R*_*T*_ > 1 if and only if *ρ*(Φ_*F*(*t*)−*V*(*t*)_(*T*)) > 1.
(iii) *R*_*T*_ < 1 if and only if *ρ*(Φ_*F*(*t*)−*V*(*t*)_(*T*)) < 1.

Thus, the disease-free periodic solution *E*_0_ for system (2) is locally asymptotically stable if *R*_*T*_ < 1 and unstable if *R*_*T*_ > 1.

Define

(17) $$\begin{array}{c} \left[\;f\;\right]≔\frac{1}{T}\overset{T}{\underset{0}{\int }}f\left(\;t\;\right)dt \end{array}$$

as the average for a continuous periodic function *f*(*t*) with the period *T*. Let [*R*_*T*_] be the basic reproduction number of the autonomous systems obtained from the average of system (2); that is,

(18) S′=Λ−βSI−μS,L′=1−pβSI−γL−μL,I′=pβSI+γL−σI−αI−μI.

An example is given to show that the basic reproduction number of the time-averaged autonomous systems may underestimate, estimate, or overestimate the infection risk.

**Example 4 .**

Consider the following:

(19) βt=b01+k1cos⁡πt+1T/2,γt=g01+k2cos⁡πt−1T/2,αt=a01+k3cos⁡πt−1T/2.

Now Lemma 2 is applied to calculate the basic reproduction number *R*_*T*_ of system (2).

**Figure 5:**
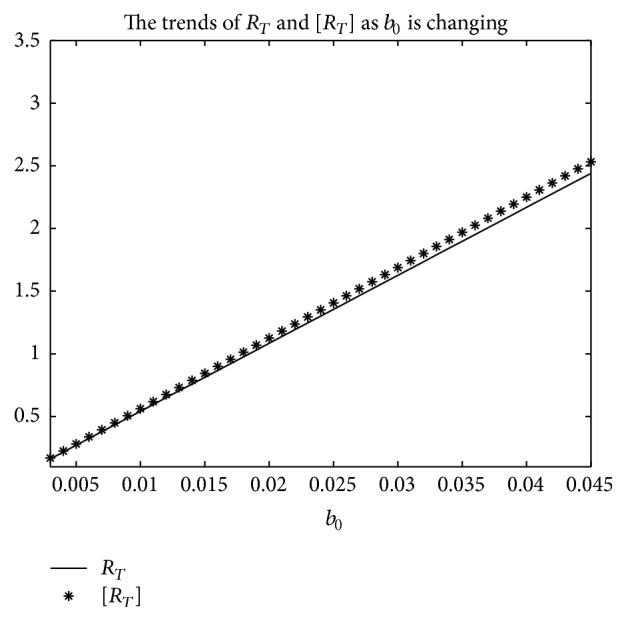
For system (2), the graph of the average basic reproduction number [*R*_*T*_] and the basic reproduction number *R*_*T*_ with respect to *b*_0_ which varies from 0.003 to 0.045, and Λ = 0.8, *μ* = 0.008, *p* = 0.08, *g*_0_ = 0.003, *k*_1_ = *k*_2_ = *k*_3_ = 1, *σ* = 0.5, *T* = 12, and *a*_0_ = 0.08.

**Figure 6:**
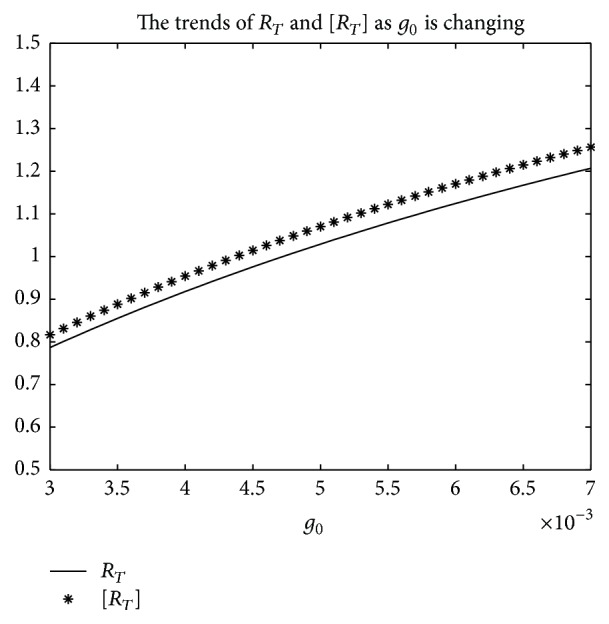
For system (2), the graph of [*R*_*T*_] and *R*_*T*_ with respect to *g*_0_ which varies from 0.003 to 0.007 when *b*_0_ = 0.015, and other parameter values are the same as those of Figure 5.

**Figure 7:**
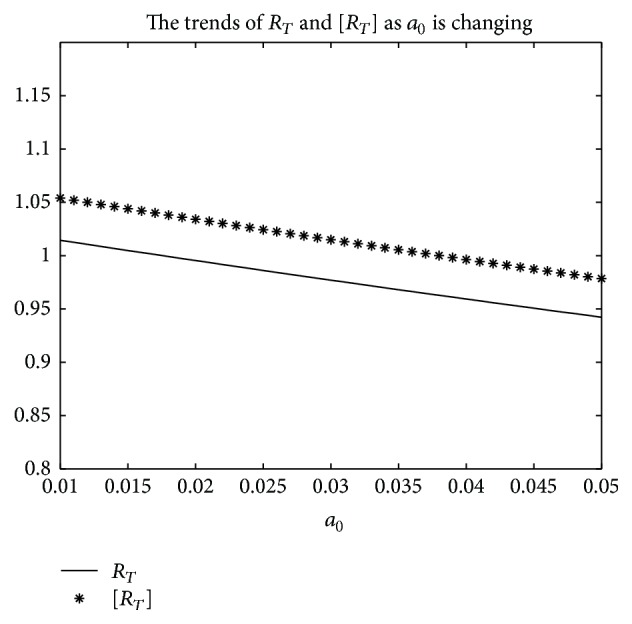
For system (2), the graph of [*R*_*T*_] and *R*_*T*_ with respect to *a*_0_ which varies from 0.01 to 0.05 when *b*_0_ = 0.0165, and other parameter values are the same as those of Figure 5.

Firstly, Λ = 0.8, *μ* = 0.008, *p* = 0.08, *g*_0_ = 0.003, *k*_1_ = *k*_2_ = *k*_3_ = 1, *σ* = 0.5, *T* = 12, and *a*_0_ = 0.08 in system (2); by numerical computation, it can acquire the curves of the average basic reproduction number [*R*_*T*_] and the basic reproduction number *R*_*T*_ when *b*_0_ varies, respectively, in Figure 5. It can be seen that [*R*_*T*_] is always greater than *R*_*T*_ as *b*_0_ is ranging from 0.003 to 0.045. Secondly, when *b*_0_ = 0.015 in system (2), *g*_0_ varies from 0.003 to 0.007, and other parameters are the same as those of Figure 5; then the numerical calculations indicate [*R*_*T*_] is greater than *R*_*T*_ in Figure 6 as *g*_0_ is varying. Thirdly, *b*_0_ = 0.015 in system (2), *a*_0_ varies from 0.01 to 0.05, and other parameters are the same as those of Figure 5; then the numerical calculations indicate [*R*_*T*_] is greater than *R*_*T*_ in Figure 7 as *a*_0_ is varying. Summing up the above, Figures 5, 6, and 7 imply that the risk of infection may be overestimated, if the average basic reproduction number [*R*_*T*_] is used.

**Figure 8:**
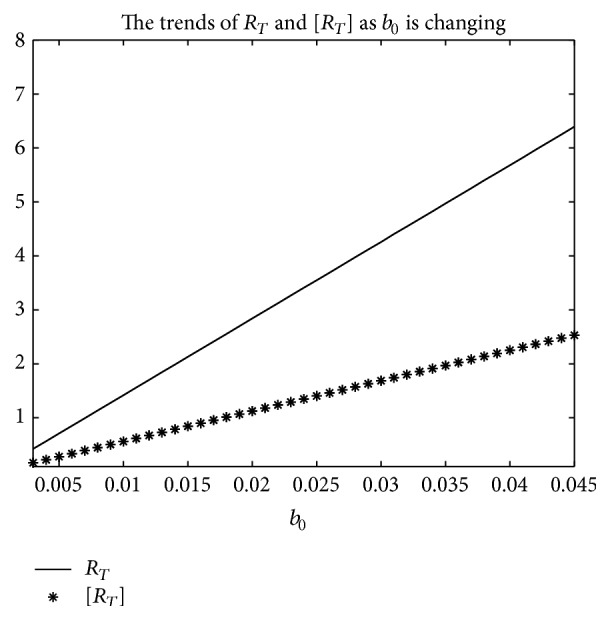
For system (2), the graph of [*R*_*T*_] and *R*_*T*_ with respect to *b*_0_ which varies from 0.003 to 0.045 when *T* = 1, and other parameter values are the same as those of Figure 5.

**Figure 9:**
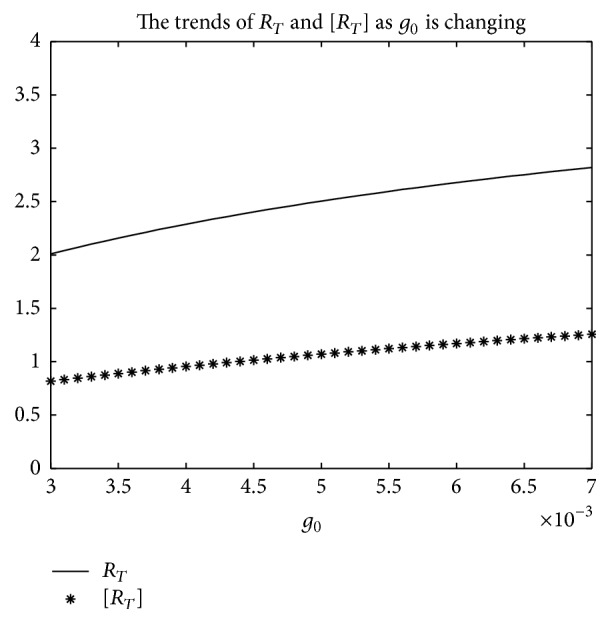
For system (2), the graph of [*R*_*T*_] and *R*_*T*_ with respect to *g*_0_ which varies from 0.003 to 0.007 when *T* = 1, and other parameter values are the same as those of Figure 6.

**Figure 10:**
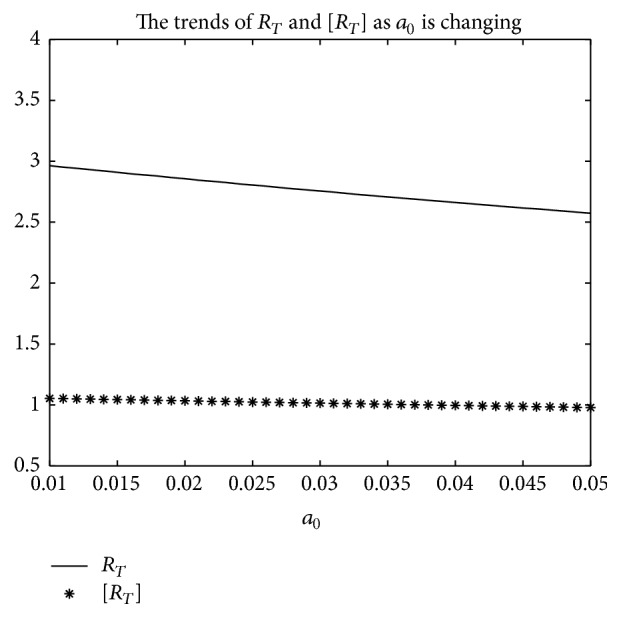
For system (2), the graph of [*R*_*T*_] and *R*_*T*_ with respect to *a*_0_ which varies from 0.01 to 0.05 when *T* = 1, and other parameter values are the same as those of Figure 7.

But, on the other hand, if *T* = 1, from Figures 8, 9, and 10, the numerical calculations indicate [*R*_*T*_] is less than *R*_*T*_ in Figures 8, 9, and 10, respectively. These imply that the risk of infection may be underestimated, if the average basic reproduction number [*R*_*T*_] is used.

**Figure 11:**
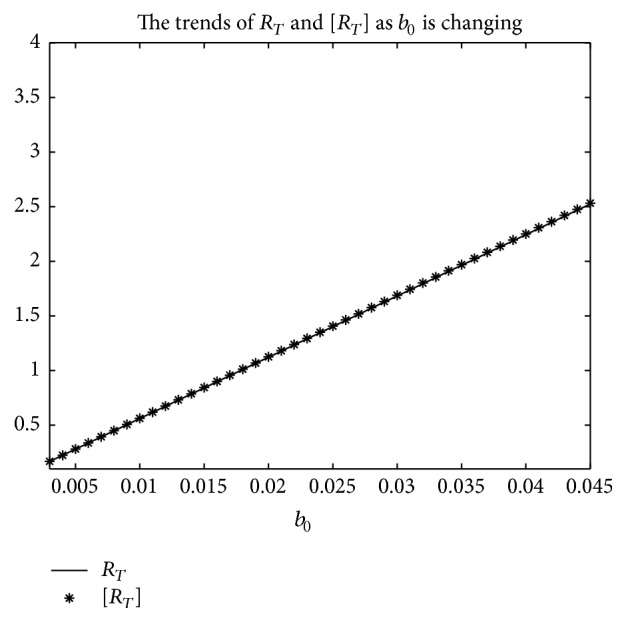
For system (2), the graph of [*R*_*T*_] and *R*_*T*_ with respect to *b*_0_ which varies from 0.003 to 0.045 when *T* = 5, and other parameter values are the same as those of Figure 5.

**Figure 12:**
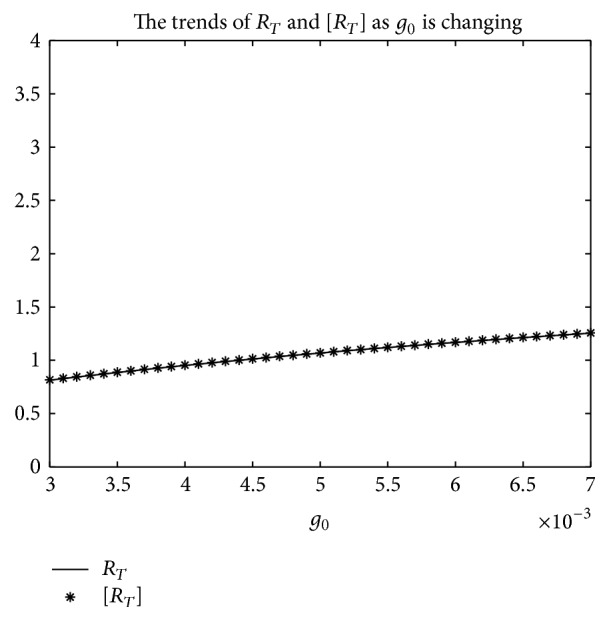
For system (2), the graph of [*R*_*T*_] and *R*_*T*_ with respect to *g*_0_ which varies from 0.003 to 0.007 when *T* = 5, and other parameter values are the same as those of Figure 6.

**Figure 13:**
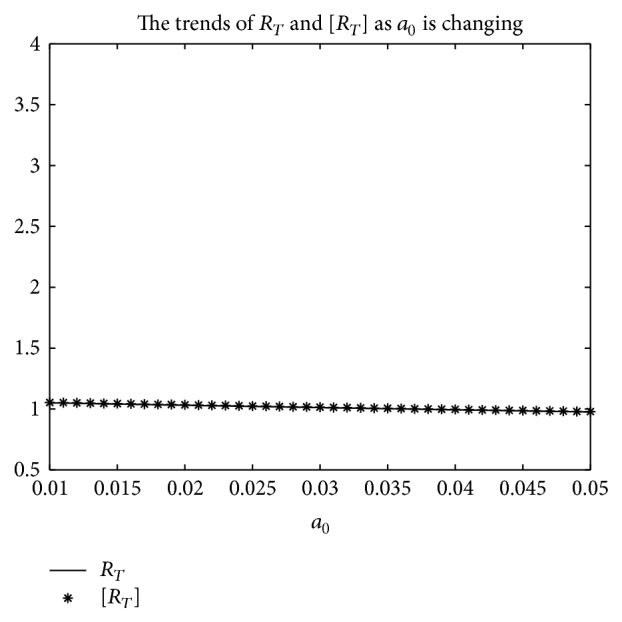
For system (2), the graph of [*R*_*T*_] and *R*_*T*_ with respect to *a*_0_ which varies from 0.01 to 0.05 when *T* = 5, and other parameter values are the same as those of Figure 7.

Especially, if *T* = 5, from Figures 11, 12, and 13, the numerical calculations indicate [*R*_*T*_] is almost equal to *R*_*T*_ in Figures 11, 12, and 13, respectively. These imply that the risk of infection may be estimated by the average basic reproduction number [*R*_*T*_] and [*R*_*T*_] can be used in some conditions.

**Figure 14:**
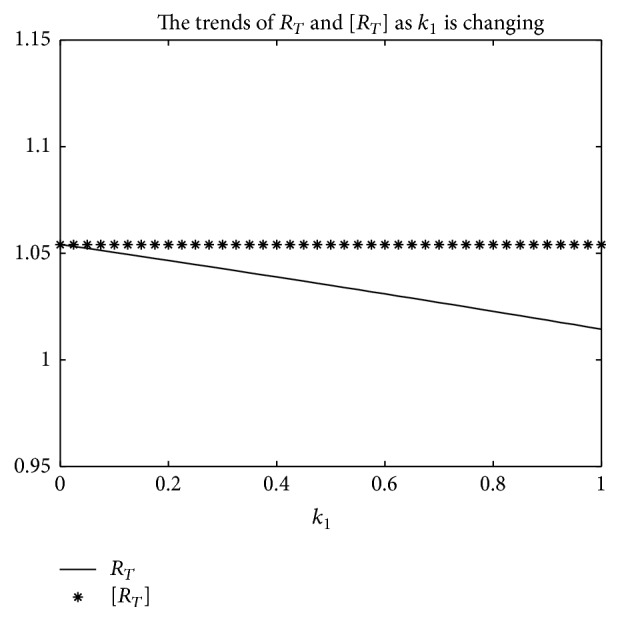
For system (2), the graph of [*R*_*T*_] and *R*_*T*_ with respect to *k*_1_ which varies from 0 to 1 when *b*_0_ = 0.0165, and other parameter values are the same as those of Figure 5.

**Figure 15:**
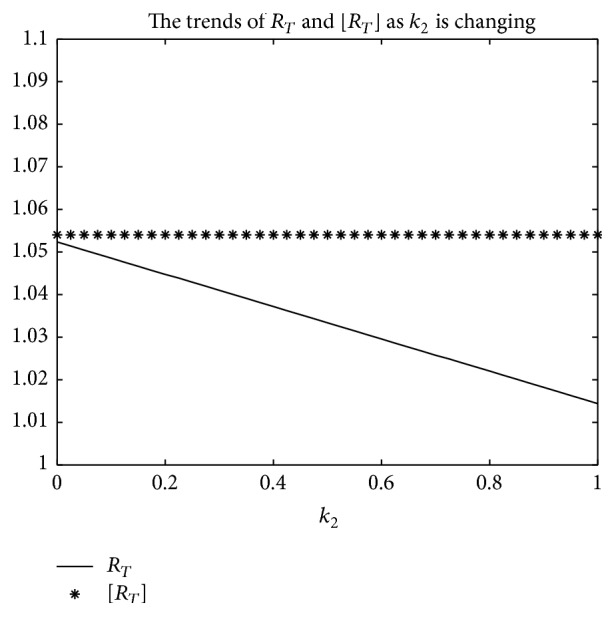
For system (2), the graph of [*R*_*T*_] and *R*_*T*_ with respect to *k*_2_ which varies from 0 to 1 when *b*_0_ = 0.0165 and other parameter values are the same as those of Figure 5.

**Figure 16:**
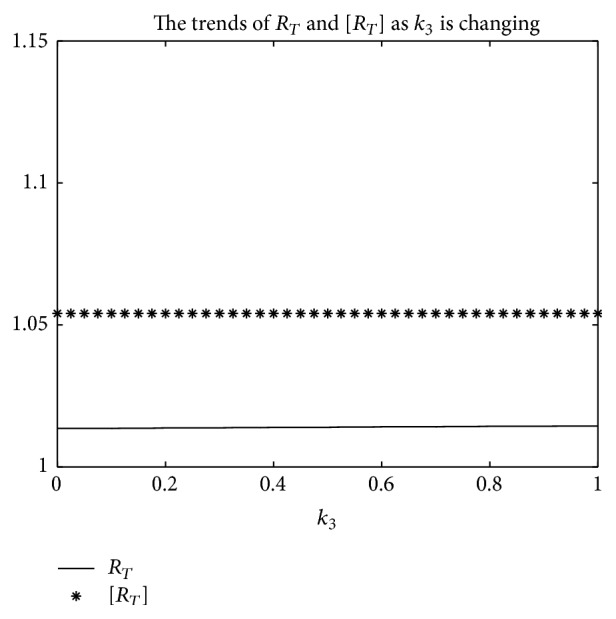
For system (2), the graph of [*R*_*T*_] and *R*_*T*_ with respect to *k*_3_ which varies from 0 to 1, when *b*_0_ = 0.0165 and other parameter values are the same as those of Figure 5.

If *b*_0_ = 0.0165 and *k*_1_, *k*_2_, *k*_3_ vary in [0,1] for system (2), respectively, with other parameters unchanged as Figure 5, numerical computation indicates (see Figures 14, 15, and 16) the average basic reproduction number overestimates the disease transmission risk.

**Figure 17:**
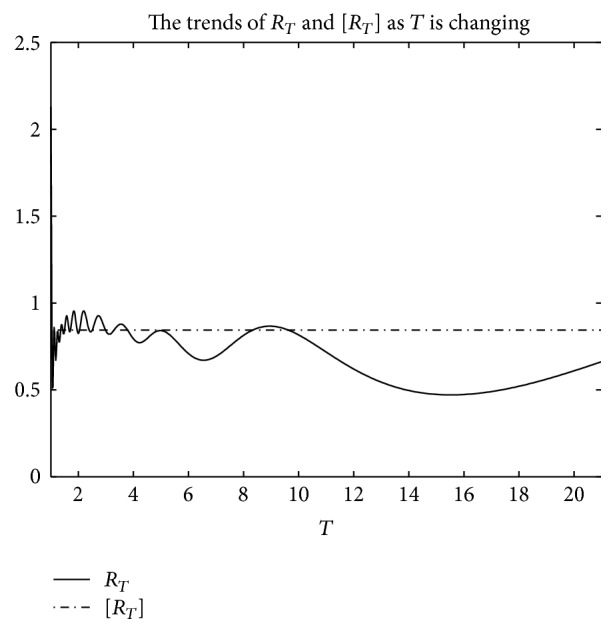
For system (2), the graph of [*R*_*T*_] and *R*_*T*_ with respect to *T* which varies from 0 to 22 when *b*_0_ = 0.015, and other parameter values are the same as those of Figure 5.

Finally, if *b*_0_ = 0.015 and *T* varies in [0,22] for system (2), with other parameters unchanged as those shown in Figure 5, numerical computation gives the relation between the basic reproduction number *R*_*T*_ and period *T* in Figure 17, which indicates the average basic reproduction number [*R*_*T*_] may be superior, inferior, or equal to the basic reproduction number *R*_*T*_, which is up to value of period *T*.

Furthermore, in the next section, we will prove some theoretical results of system (2), in which *R*_*T*_ serves as a threshold parameter: if *R*_*T*_ < 1, then there exists a globally asymptotically stable disease-free periodic state *E*_0_(Λ/*μ*, 0,0); if *R*_*T*_ > 1, then the disease is persistent in the population and there exists at least one positive periodic solution.

## 4. Extinction and Uniform Persistence

The following lemma is useful for our discussion in this section.

**Lemma 5 (see [19], Lemma 2.1).**

Let *l* = (1/*T*)ln⁡(*ρ*(Φ_*A*_(*T*))), and then there exists a positive *T*-periodic function *v*(*t*) such that *e* ^*lt*^ *v*(*t*) is a solution of (5).

**Theorem 6 .**

For system (2), the disease-free periodic state *E*_0_(Λ/*μ*, 0,0) is globally stable on set *Ω* if *R*_*T*_ < 1; and it is unstable if *R*_*T*_ > 1.

**Proof**

By Lemma 3, if *R*_*T*_ > 1, then *E*_0_(Λ/*μ*, 0,0) is unstable; and if *R*_*T*_ < 1, then *E*_0_ is locally asymptotically stable. Hence, we only need to prove that *E*_0_ is globally attractive for *R*_*T*_ < 1.

Since *S*(*t*), *L*(*t*), *I*(*t*) is a nonnegative solution of system (2) in *Ω*, we have *S* ≤ Λ/*μ*, and know that

(20) L′≤1−pβtΛμI−γtL−μL,I′≤pβtΛμI+γtL−σI−αtI−μI,

for *t* ≥ 0.

Consider an auxiliary system:

(21) L~′=1−pβtΛμI~−γtL~−μL~,I~′=pβtΛμI~+γtL~−σI~−αtI~−μI~;

that is,

(22) $$\begin{array}{c} {\left(\;\begin{array}{c} \tilde{L}\\ \tilde{I} \end{array}\;\right)}^{\prime }=\left(\;F\left(\;t\;\right)-V\left(\;t\;\right)\;\right)\left(\begin{array}{c} \tilde{L}\\ \tilde{I} \end{array}\right). \end{array}$$

It follows from Lemma 5 that there exists a positive *T*-periodic function *v*_1_(*t*), such that *e* ^*l*_1_*t*^ *v*_1_(*t*) is a solution of (22), where *l*_1_ = (1/*T*)ln⁡(*ρ*(Φ_*F*(*t*)−*V*(*t*)_(*T*))). Choose *t*_1_ ≥ 0 and a real number *a*_1_ > 0 such that

(23) $$\begin{array}{c} \left(\begin{array}{c} L\left(t_{1}\right)\\ I\left(t_{1}\right) \end{array}\right)\leq a_{1}v_{1}\left(\;0\;\right). \end{array}$$

By the comparison principle, we get

(24) $$\begin{array}{c} \left(\begin{array}{c} L\left(t\right)\\ I\left(t\right) \end{array}\right)\leq a_{1}v_{1}\left(\;t-t_{1}\;\right)e^{l_{1}\left(t-t_{1}\right)},\;\forall t\geq t_{1}. \end{array}$$

By Lemma 3, it is easy to know that *R*_*T*_ < 1 if and only if *ρ*(Φ_*F*(*t*)−*V*(*t*)_(*T*)) < 1, thus *l*_1_ = (1/*T*)ln⁡(*ρ*(Φ_*F*(*t*)−*V*(*t*)_(*T*))) < 0. Therefore, *L*(*t*) → 0, *I*(*t*) → 0, and *S*(*t*) → Λ/*μ* as *t* → *∞*; that is, *E*_0_(Λ/*μ*, 0,0) is globally attractive for *R*_*T*_ < 1. In conclusion, *E*_0_ is globally asymptotically stable if *R*_*T*_ < 1.

**Theorem 7 .**

If *R*_*T*_ > 1, system (2) is uniformly persistent, and there exists at least one positive periodic solution.

**Proof**

Denote *Ω*_0_≔{(*S*, *L*, *I*) ∈ *Ω* : *L* > 0,  *I* > 0} and ∂*Ω*_0_≔*Ω*∖*Ω*_0_. And then *x*_0_ = (*S*_0_, *L*_0_, *I*_0_) ∈ *Ω*_0_. Let *P* : *Ω* → *Ω* be the Poincaré map associated with system (2); that is, *P*(*x*_0_) = *u*(*T*, *x*_0_), ∀*x*_0_ ∈ *Ω*, where *φ*(*t*, *x*_0_) is the unique solution of system (2) with *φ*(0, *x*_0_) = *x*_0_.

Now it is proved that *P* is uniformly persistent with respect to (*Ω*_0_, ∂*Ω*_0_).

It is easy to see that *Ω* and *Ω*_0_ are positively invariant, ∂*Ω*_0_ is a relatively closed set in *Ω*, and *P* is point dissipative from Theorem 1.

Set *M*_∂_ = {(*S*_0_, *L*_0_, *I*_0_)∈∂*Ω*_0_ : *P* ^*m*^(*S*_0_, *L*_0_, *I*_0_)∈∂*Ω*_0_,  ∀*m* ≥ 0}.

We claim that

(25) $$\begin{array}{c} M_{\partial }=\left\{\;\left(\;S,0,0\;\right):S\geq 0\;\right\}. \end{array}$$

Obviously, {(*S*, 0,0) : *S* ≥ 0}⊆*M*_∂_. For any (*S*_0_, *L*_0_, *I*_0_) ∈ ∂*Ω*_0_∖{(*S*, 0,0) : *S* ≥ 0}, if *L*_0_ > 0, *I*_0_ = 0, then *L*(*t*) > 0, ∀*t* ≥ 0, and then *I*′ = *γ*(*t*)*L* > 0. For the other case, *L*_0_ = 0, *I*_0_ > 0, and then *I*(*t*) > 0, and, from the first equation of system (2), thus

(26) Ste−∫0tβsIs+μdsS0+Λ∫0te∫0sβζIζ+μdζds≥Λe−∫0tβsIs+μds∫0te∫0sβζIζ+μdζds>0,

for any *t* > 0.

From the second equation of system (2), we have

(27) Lt=e−∫0tγs+μdsL0+∫0t1−pβsSsIse∫0sγζ+μdζds>0,∀t>0.

It then follows that $(S(t),L(t),I(t))\;\bar{\in }\;\partial \Omega_{0}$ for 0 < *t* ≪ 1. Thus, the positive invariance of *Ω*_0_ implies (25).

Clearly, there is a unique fixed point of *P* in *M*_∂_, which is *E*_0_(Λ/*μ*, 0,0).

For system (2), by the continuity solutions with respect to the initial values, ∀*α* > 0, there exists *α* ^*∗*^ > 0 such that, for all *x*_0_ ∈ *Ω*_0_ with ‖*x*_0_ − *E*_0_‖ ⩽ *α* ^*∗*^, we have ‖*ϕ*(*t*, *x*_0_) − *ϕ*(*t*, *E*_0_)‖ < *α*, ∀*t* ∈ [0, *T*].

Then, we will show that

(28) $$\begin{array}{c} \underset{m\to \infty }{\mathrm{limsup}}\;d\left(\;P^{m}\left(\;x_{0}\;\right),E_{0}\;\right)\geq \alpha^{*},\;\forall x_{0}\in \Omega_{0}. \end{array}$$

If not, then

(29) $$\begin{array}{c} \underset{m\to \infty }{\mathrm{limsup}}\;d\left(\;P^{m}\left(\;x_{0}\;\right),E_{0}\;\right)<\alpha^{*} \end{array}$$

for some *x*_0_ ∈ *Ω*_0_.

Without loss of generality, we can assume that

(30) $$\begin{array}{c} d\left(\;P^{m}\left(\;x_{0}\;\right),E_{0}\;\right)<\alpha^{*},\;\forall m\geq 0. \end{array}$$

Then, we have

(31) ϕt,Pmx0−ϕt,E0<α,∀m≥0,  ∀t∈0,T.

For any *t* ≥ 0, let *t* = *mT* + *t*_1_, where *t*_1_ ∈ [0, *T*) and *m* is the largest integer less than or equal to *t*/*T*. Therefore, we have

(32) ϕt,Pmx0−ϕt,E0=ϕt1,Px0−ϕt1,E0<α,∀t≥0.

Note that *x*(*t*)≔(*S*(*t*), *L*(*t*), *I*(*t*)) = *ϕ*(*t*, *x*_0_). It then follows that 0 ≤ *S*(*t*), *L*(*t*), *I*(*t*) ≤ *α*, ∀*t* ≥ 0. From the first equation of system (2), we have

(33) $$\begin{array}{c} S^{\prime }\geq \Lambda -\beta \left(\;t\;\right)S\alpha -\mu S. \end{array}$$

Note that the perturbed system

(34) $$\begin{array}{c} {\hat{S}}^{\prime }=\Lambda -\beta \left(\;t\;\right)\hat{S}\alpha -\mu \hat{S} \end{array}$$

admits a unique positive *T*-periodic solution

(35) S^t,α=e−∫0tβsα+μdsS^0,α+Λ∫0te∫0sβζα+μdζds

which is globally attractive in *ℝ*_+_, where

(36) $$\begin{array}{c} \hat{S}\left(\;0,\alpha \;\right)=\frac{\Lambda \int_{0}^{T}e^{\int_{0}^{s}\left(\beta \left(\zeta \right)\alpha +\mu \right)d\zeta }ds}{1-e^{\int_{0}^{T}\left(\beta \left(s\right)\alpha +\mu \right)ds}}>0. \end{array}$$

Applying Lemma 3, we know that *R*_*T*_ > 1 if and only if *ρ*(Φ_*F*(*t*)−*V*(*t*)_(*T*)) > 1. By continuity of the spectrum for matrices ([20], Section II. 5.8.), we can choose *η*, which is small enough, such that *ρ*(*ϕ*_*F*(*t*)−*V*(*t*)−*ηM*(*t*)_(*T*)) > 1, where

(37) $$\begin{array}{c} M\left(\;t\;\right)=\left(\begin{array}{cc} 0 & \left(1-p\right)\beta \left(t\right)\\ 0 & p\beta \left(t\right) \end{array}\right). \end{array}$$

Since $\hat{S}(0,\alpha )$ is continuous in *α*, we can fix *α* > 0 small enough that $\hat{S}(t,\alpha )>\hat{S}(t)-\eta$, ∀*t* ≥ 0. Furthermore, since the fixed point $\hat{S}(0,\alpha )$ of the Poincaré map associated with (34) is globally attractive, there exists $\hat{t}>0$ such that $S(t)>\hat{S}(t)-\eta$ for $t\geq \hat{t}$. As a consequence, for $t\geq \hat{t}$, it holds that

(38) L′≥1−pβtS^t−ηI−γt+μL,I′≥pβtS^t−ηI+γtL−σ+αt+μI.

Consider another auxiliary system

(39) L~′=1−pβtS^t−ηI~−γt+μL~,I~′=pβtS^t−ηI~+γtL~−σ+αt+μI~.

It follows from Lemma 5 that there exists a positive *T*-periodic function $\left(\tilde{L}(t),\tilde{I}(t)\right)$ such that $\left(\tilde{L}(t),\tilde{I}(t))=e^{lt}(\tilde{L}(t),\tilde{I}(t)\right)$ is a solution of (39), where

(40) $$\begin{array}{c} l=\frac{1}{T}\ln\left(\;\rho \left(\;\phi_{F\left(t\right)-V\left(t\right)-\eta M\left(t\right)}\left(\;T\;\right)\;\right)\;\right). \end{array}$$

Choose $\bar{t}\geq \hat{t}$ and a small *α*_2_ > 0 such that $\left(L(\bar{t}),I(\bar{t}))\geq (\tilde{L}(0),\tilde{I}(0)\right)$. By the comparison principle we get $(L(t),I(t))\geq \alpha_{2}(\tilde{L}(t-\bar{t}),\tilde{I}(t-\bar{t}))e^{l(t-\bar{t})}$, $\forall t\geq \bar{t}$. Since *R*_*T*_ > 1, *ρ*(*ϕ*_*F*(*t*)−*V*(*t*)−*ηM*(*t*)_(*T*)) > 1. And thus *l* > 0, which implies that *L*(*t*) → *∞* and *I*(*t*) → *∞* as *t* → *∞*. This leads to a contradiction.

So suppose (29) is wrong; that is, (28) is right. Furthermore, (28) shows that *E*_0_ is an isolated invariant set in *Ω*, and *W* ^*s*^(*E*_0_)∩*Ω*_0_ = Φ. Every orbit in *M*_∂_ converges to *E*_0_, and *E*_0_ is acyclic in *M*_∂_. By the acyclicity theorem on uniform persistence for maps ([21], Theorem 1.3.1 and Remark 1.3.1), it follows that *P* is uniformly persistent with respect to (*Ω*_0_, ∂*Ω*_0_). Thus ([21], Theorem 3.1.1) implies the uniform persistence of the solutions of system (2) with respect to (*Ω*_0_, ∂*Ω*_0_); that is, there exists *ε* > 0 such that any solution (*S*(*t*), *E*(*t*), *I*(*t*)) of (2) with initial values (*S*(0), *E*(0), *I*(0)) ∈ *Ω*_0_ satisfies lim_*t*→*∞*_ *L*(*t*) ≥ *ε* and lim_*t*→*∞*_ *I*(*t*) ≥ *ε*. Moreover, by Zhao ([21], Theorem 1.3.6), *P* has a fixed point (*S* ^*∗*^(0), *E* ^*∗*^(0), *I* ^*∗*^(0)) ∈ *Ω*_0_. From the first equation of (2), *S* ^*∗*^(*t*) satisfies *S* ^*∗*^′ ≥ *μA* − *β*(*t*)*S* ^*∗*^Λ/*μ* − *μS* ^*∗*^. By the comparison theorem, we have *S* ^*∗*^ ≥ *e* ^−∫_0_^*t*^(*β*(*s*)Λ/*μ*+*μ*)*ds*^(*S* ^*∗*^(0) + Λ∫_0_^*t*^ *e* ^∫_0_^*s*^(*β*(*τ*)Λ/*μ*+*μ*)*dτ*^ *ds*) > Λ*e* ^−∫_0_^*t*^(*β*(*s*)Λ/*μ*+*μ*)*ds*^∫_0_^*t*^ *e* ^∫_0_^*s*^(*β*(*τ*)Λ/*μ*+*μ*)*dτ*^ *ds* > 0,   ∀*t* > 0. The seasonality of *S* ^*∗*^(*t*) implies *S* ^*∗*^(0) > 0. By the second and third equations of (2) and the irreducibility of the cooperative matrix

(41) $$\begin{array}{c} \left(\begin{array}{cc} -\left(\gamma \left(t\right)+\mu \right) & \left(1-p\right)\beta \left(t\right)S^{*}\left(t\right)\\ \gamma \left(t\right) & p\beta \left(t\right)S^{*}\left(t\right)-\left(\sigma +\alpha \left(t\right)+\mu \right) \end{array}\right), \end{array}$$

it follows that $(L^{*}(t),I^{*}(t))\in \underset{}{\mathrm{Int}}\left({\mathbb{R}}_{+}^{2}\right)$, ∀*t* ≥ 0. Consequently, (*S* ^*∗*^(*t*), *L* ^*∗*^(*t*), *I* ^*∗*^(*t*)) is a positive *T*-periodic solution of (2).

**Figure 18:**
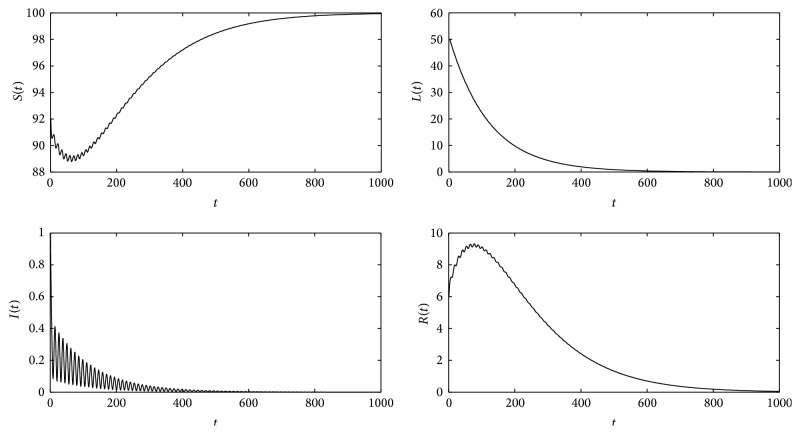
For system (2), *β*(*t*), *γ*(*t*), and *α*(*t*) are listed in Example 4. Λ = 0.8, *μ* = 0.008, *p* = 0.08, *a*_0_ = 0.08, *g*_0_ = 0.003, *k*_1_ = *k*_2_ = *k*_3_ = 1, *σ* = 0.5, *T* = 12, and *b*_0_ = 0.005, and then *R*_*T*_ = 0.2814. These figures show that the disease will die out, which is the same as Theorem 6.

**Figure 19:**
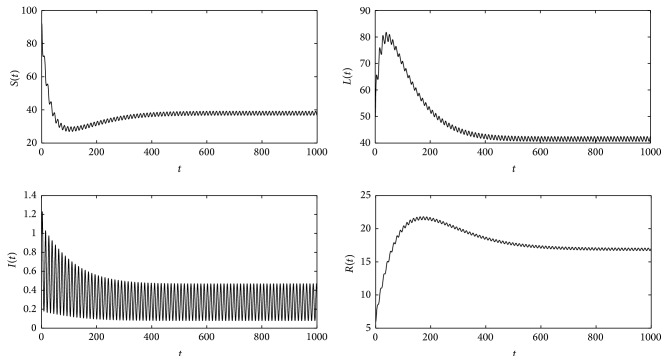
For system (2), *b*_0_ = 0.04 and other parameter values are the same as those of Figure 18; then *R*_*T*_ = 2.251. These figures show that the disease will be asymptotic to a periodic solution, which is the same as Theorem 7.

Theorems 6 and 7 have shown that *R*_*T*_ is a threshold parameter which determines whether or not the disease persists in the population. Now, some numerical simulations (Figures 18 and 19) are presented to demonstrate these results. And in these simulations, *T* = 12, according to the fact that by one year has 12 months. In Figures 18 and 19, the simulations verify Theorems 6 and 7, respectively.

## 5. Discussion

Monthly pulmonary TB cases, from January 2004 to December 2012 in Shaanxi province, have been analyzed by the seasonal adjustment program. It has been found that TB cases show seasonal variation in Shaanxi: the peak months (January and March) compare to the lowest trough month (December). It is necessary to study the seasonality TB epidemic model according to the seasonal component of Shaanxi's data. Considering the regularity of peak TB seasonality may help allocate resources for the prevention and treatment of TB activities, a periodic TB epidemic model has been formulated and studied.

The basic reproduction number *R*_*T*_ for the periodic model is given. By numerical simulation, *R*_*T*_ has been compared to the average basic reproduction number [*R*_*T*_] in different parameter values. It has shown that [*R*_*T*_] may overestimate or underestimate *R*_*T*_ or be equal to *R*_*T*_ in different cases. It is also up to the value of periodic *T*. Furthermore, in theory, the threshold dynamics has been studied for the periodic TB model: if *R*_*T*_ < 1, then the disease-free equilibrium is globally asymptotically stable; that is, the TB disease will disappear eventually; if *R*_*T*_ > 1, then there exists at least one positive periodic solution and the disease will be uniformly persistent.

Our numerical simulations are in good accordance with these theoretical results. It should be noted that we have not fit Shaanxi's data in these simulations since we cannot accurately estimate some parameters' values in the periodic model according to available data or references. Despite lack of comparing the model results with the Shaanxi's data, our theoretical results have shown that the basic reproduction number with periodic *R*_*T*_ plays a crucial role in determining dynamics of the seasonality TB disease and could be used for controlling the spread of TB epidemic in reality strategies.

## Figures and Tables

**Table 1:** Shaanxi TB cases month report from January 2004 to December 2012 [12].

Month/year	2004	2005	2006	2007	2008	2009	2010	2011	2012
January	2761	3781	3727	3751	3250	2696	2586	2290	2309
February	3057	2613	3118	2914	3033	2486	2038	2187	2440
March	3519	4865	3774	3178	3531	2956	2382	2489	2531
April	3284	4739	3254	2996	3005	2606	2435	2260	2276
May	3064	3602	3064	2909	3182	2556	2314	2325	2258
June	3008	3514	2660	2812	2872	2343	2148	2173	1975
July	3076	3142	2644	2508	2674	2317	2091	1957	1923
August	2822	3293	2569	2480	2540	2234	2076	2061	1878
September	2465	3121	2248	2317	2478	2229	1998	1969	1651
October	2517	2554	2328	2204	1602	2133	2036	1966	1662
November	2306	2653	1721	1989	1954	2021	1849	1837	1595
December	1580	1946	1300	1352	1581	1953	1662	1799	1548

Sum	33459	39823	32407	31410	32702	28530	25615	25313	24046
